# Based on Network Pharmacology and Gut Microbiota Analysis to Investigate the Mechanism of the Laxative Effect of Pterostilbene on Loperamide-Induced Slow Transit Constipation in Mice

**DOI:** 10.3389/fphar.2022.913420

**Published:** 2022-05-16

**Authors:** Zhiwei Yao, Siqi Fu, Bingbing Ren, Lushun Ma, Daqing Sun

**Affiliations:** Department of Pediatric Surgery, Tianjin Medical University General Hospital, Tianjin, China

**Keywords:** pterostilbene, slow transit constipation, network pharmacology, gut microbiota, oxidative stress, interstitial cells of cajal

## Abstract

**Background:** Pterostilbene (PTE) is a natural polyphenol compound that has been proven to improve intestinal inflammation, but its laxative effect on slow transit constipation (STC) has never been studied. This study aims to investigate the laxative effect of PTE on loperamide (LOP)-induced STC mice and its influence on intestinal microbes through a combination of network pharmacological analysis and experimental verification.

**Material and Methods:** PTE was used to treat LOP-exposed mice, and the laxative effect of PTE was evaluated by the total intestinal transit time and stool parameters. The apoptosis of Cajal interstitial cells (ICCs) was detected by immunofluorescence. The mechanism of PTE’s laxative effect was predicted by network pharmacology analysis. We used western blot technology to verify the predicted hub genes and pathways. Malondialdehyde (MDA) and GSH-Px were tested to reflect oxidative stress levels and the changes of gut microbiota were detected by 16S rDNA high-throughput sequencing.

**Results:** PTE treatment could significantly improve the intestinal motility disorder caused by LOP. Apoptosis of ICCs increased in the STC group, but decreased significantly in the PTE intervention group. Through network pharmacological analysis, PTE might reduce the apoptosis of ICCs by enhancing PI3K/AKT and Nrf2/HO-1 signaling, and improve constipation caused by LOP. In colon tissues, PTE improved the Nrf2/HO-1 pathway and upregulated the phosphorylation of AKT. The level of MDA increased and GSH-Px decreased in the STC group, while the level of oxidative stress was significantly reduced in the PTE treatment groups. PTE also promoted the secretion of intestinal hormone and restored the microbial diversity caused by LOP.

**Conclusion:** Pterostilbene ameliorated the intestinal motility disorder induced by LOP, this effect might be achieved by inhibiting oxidative stress-induced apoptosis of ICCs through the PI3K/AKT/Nrf2 signaling pathway.

## 1 Introduction

Functional constipation (FC) is a common gastrointestinal dysfunction symptom around the world. In recent years, the incidence has been rising rapidly (Shin et al., 2019) (van Mill et al., 2019). Epidemiological data showed that the percentage of FC in adults is as high as 15.3% (Barberio et al., 2021). Slow transit constipation (STC), which accounts for about 55% of FC (Tanner et al., 2021), It is main clinical features are slowing of intestinal motility and delayed passage of intestinal contents. Senna and other laxatives often appear in the treatment of STC. However, long-term administration of these agents does not provide satisfactory efficacy and severe adverse effects including drug dependence and melanosis are noted in patients (Bassotti and Blandizzi, 2014) (Lim et al., 2019). Therefore, it is necessary to explore safe and effective alternative treatment options.

Interstitial cells of Cajal (ICCs) are pacemaker cells for many rhythmic movements in the gastrointestinal tract (Horváth et al., 2006). The number of ICCs in the colon of STC patients is significantly reduced (He et al., 2000) (Serra et al., 2020), and ICCs defects have also been consistently found in animal models of STC (He et al., 2000) (Zhu et al., 2016). Further research found that the network of ICCs also undergoes periodic changes under normal physiological conditions (Gibbons et al., 2009). Apoptosis can lead to loss of ICCs, and was observed in a large proportion of STC patient colons (Zheng et al., 2021) (Faussone-Pellegrini, 2005). Therefore, maintenance of ICCs function has been regarded as an important therapeutic method for STC.

Elevated levels of oxidative stress have been found in both constipated patients and animal models (Wang et al., 2004) (Lee et al., 2020). At the same time, some studies have found that the increased apoptosis of ICCs in diabetic gastroparesis mice is related to oxidative stress (Tian et al., 2018) (Wang et al., 2021c). However, the relationship between ICCs apoptosis and oxidative stress in STC animal models has not been reported.

**FIGURE 1:**
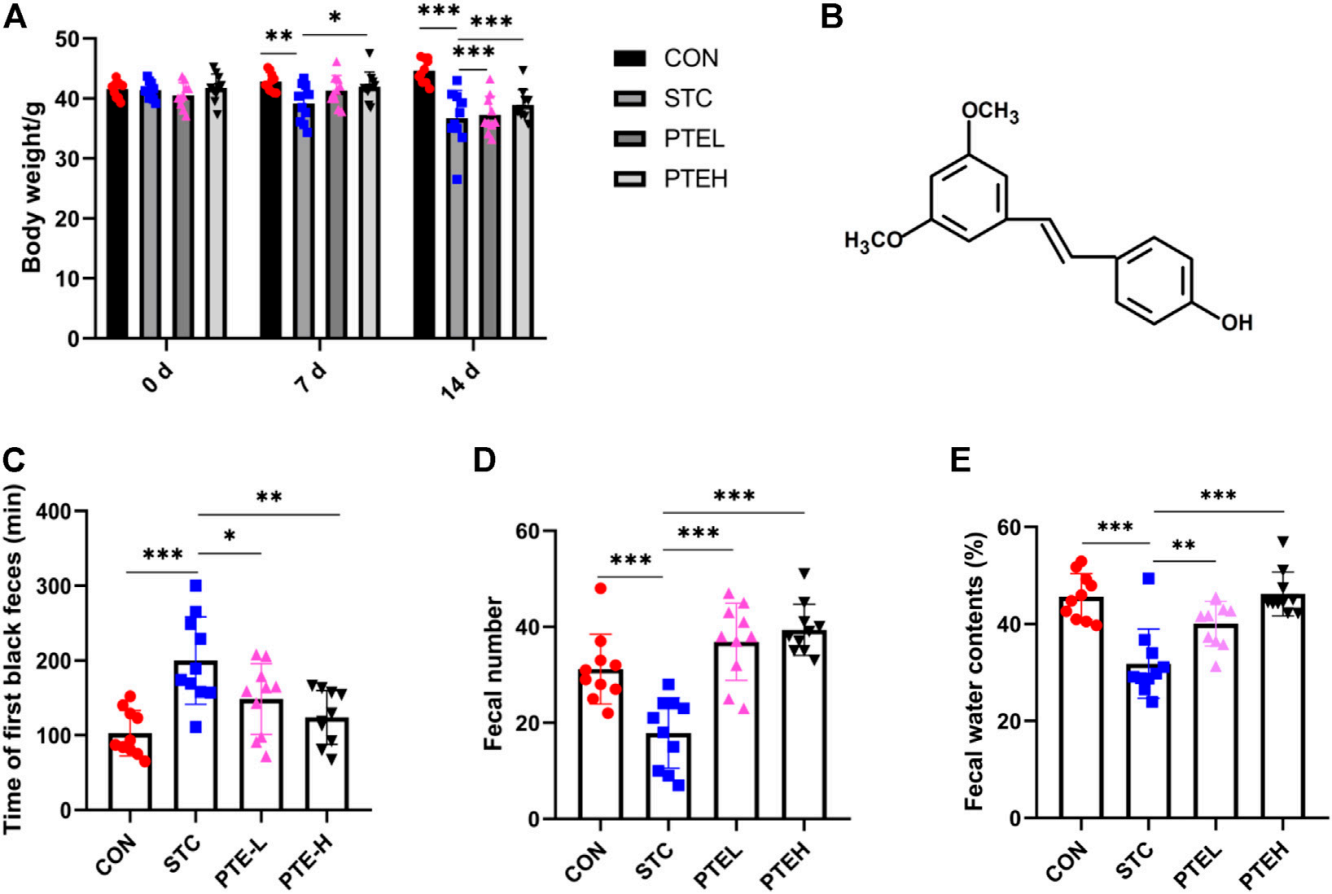
PTE alleviated LOP-induced constipation symptoms. **(A)** Body weight. **(B)** Chemical structure of PTE. Fecal parameters: **(C)** The whole gut transit time. **(D)** Fecal number. **(E)** Water contents. (*n* = 10). *p < 0.05, **p < 0.01, ***p < 0.001. (LOP, loperamide; STC, slow transit constipation; PTE, pterostilbene).

Pterostilbene (PTE) is a natural derivative of resveratrol, mainly derived from berries and grapes (chemical structure of PTE was shown in Figure 1B). It has been found to have a protective effect in colitis caused by high-fat diet and dextran sulfate sodium (Chen et al., 2021) (Fan-Jiang et al., 2021). Previous studies also found that polyphenol compounds had the effect of promoting the motility of the gut (Koh et al., 2021). In addition, PTE has also been shown to improve the disordered intestinal microflora of rats fed high-fat (Milton-Laskibar et al., 2021). However, the laxative effect and the influence of the intestinal microflora of PTE on STC mice have not been studied, and whether the apoptosis of ICCs is involved in the effect of PTE on colonic transit is also unclear.

The aim of this study was to verify the laxative effect of PTE in loperamide (LOP)-induced STC model mice and whether these therapeutic effects might be mediated by improving oxidative stress, apoptosis of ICCs and intestinal flora. We predicted the potential targets and mechanisms of PTE in the treatment of STC by network analysis, and validated the predicted results by *in vivo* experiments.

## 2 Material and Methods

### 2.1 Animals

Forty Kunming male mice (Specific Pathogen Free), 8 weeks old, weighing 41.32 ± 1.837 g, were purchased from Vital River Co., Ltd (Beijing). All animals were raised in the experimental animal center of Tianjin Medical University (constant temperature 25°C, 12 h light/dark cycle). All experimental protocols involving animals were approved by the Animal Ethics Committee of Tianjin Medical University.

### 2.2 Experiment Grouping and Model Preparation

Before the experiment, mice were randomly divided into four groups: CON (control group), STC (STC model group), PTEL (PTE low-dose group), and PTEH (PTE high-dose group). *n* = 10. Except for the CON group, the other groups were given 5 mg/kg LOP (34,014, Sigma) by gavage to replicate the mice constipation model for 7 days. The LOP suspension was prepared with physiological saline, and the administration volume was 10 ml/kg, twice daily. The CON group was given normal saline by intragastric administration with the same volume, twice a day, for 7 days. Starting from day 8, the dose of LOP was doubled to 10 mg/kg, and PTE (Macklin Biochemical Co., Ltd, P815984, Shanghai) was given 1 h after administration of LOP. PTE was prepared as a suspension with 0.5% carboxymethylcellulose sodium (CMC-Na) (C299502, Aladdin, Shanghai); the CON group and STC group were given 0.5% CMC-Na by gavage, 10 ml/kg, once a day, for 7 days. PTE groups were administered intragastrically in the low-dose group (30 mg/kg) and the high-dose group (60 mg/kg) for 7 days (Supplementary Figure S1).

### 2.3 Determination of Defecation Parameters

During the period from modeling to sampling, the mental state, fur, eating situation, stool quality, and quantity of each group of mice were observed every day. After the last dose, the mice in each group were fasted with water for 12 h and were fed with 0.2 ml India ink (S30881, Yuanye Bio-Technology, Shanghai, China). Then, all animals returned to normal food intake and were reared in a single cage. From the end of the gavage ink, recorded the time (min) to discharge the first black stool, collected the feces within 6 h, recorded the number of pellets, and weighed (wet weight A). The fecal pellets were placed in an oven at 70°C and continuously dried for 24 h and weighed (dry weight B), and the moisture content of the feces was calculated = (A-B)/A × 100%.

### 2.4 Network Pharmacology Analysis

#### 2.4.1 Screening of Pterostilbene Treatment Slow Transit Constipation Targets

PharmMapper, STITCH, SwissTargetPrediction and Chembl database were used to predict the potential target genes of PTE (Yang et al., 2020). After merging the above four database targets, the PTE-related targets was uniformly transformed by the UniPort database (Zhang Y. et al., 2021). “slow transit constipation” was used as the key words in the PharmGkb, OMIM, GeneCards, Drugbank, and TTD databases to search for STC-related targets (Yu et al., 2021). The detailed information about databases and platforms was shown in Supplementary Table S1.

PTE and STC disease target data were imported together to R software (3.6.3) and got the intersection of the drug and disease targets, the intersection genes were the potential target of PTE to treat STC, then output the venn diagram. According to the article of Liu (Zhang W. et al., 2021), the intersection genes were imported into the STRING database online to build a PPI network, set the confidence score:≥0.9, after the protein interaction analysis, the resulting file was exported by Cytoscape software (CytoNCA plug-in), and the core target map of the protein interaction network was made (Zhang W. et al., 2021).

#### 2.4.2 GO Function and Kyoto Encyclopedia of Genes and Genomes Pathway Enrichment Analysis

The common targets of disease and drug were calculated by ClusterProfiler package in R software (v3.6.3) to execute GO (gene ontology-biological process) function and KEGG (Kyoto encyclopedia of genes and genomes) pathway enrichment analysis, and *p* < 0.05 for target Gene screening, analysis of the biological processes and main signal pathways of PTE’s pharmacological effects (Li XM. et al., 2021).

### 2.5 Detection of Gut Hormones and Oxidative Stress-Related Indicators

After the last experiment, all mice were anesthetized and then blood was taken from the eyeballs, the upper serum was centrifuged (3,500 r/min, 10 min) to determine the level of motilin (MTL) and gastrin (Gas) by ELISA kit (Mlbio, Shanghai, China) and the levels of malondialdehyde (MDA) and GSH-Px were detected by available kits (A001-3 and A003-1, Nanjing Jiancheng, China).

### 2.6 Immunofluorescence

Immunofluorescence staining was performed as previously reported (Wang et al., 2021c). For Immunofluorescence staining, colon tissue paraffin sections (5 μm) were used. The slides were deparaffinized in xylene, subsequently hydrated in different concentrations of ethanol and antigen unmasked using sodium citrate buffer for 20 min. The samples were then treated with 0.2% Triton-X (Sigma) for 30 min at room temperature. Next, the sections were incubated with anti-c-kit antibody (R & D, AF1356, 1:200) overnight at 4°C. After washing three times in PBS, the slides were incubated with secondary antibodies (1:200) for 1 h at 37°C. In order to detect the apoptosis of ICCs, the TUNEL kit (Roche, Germany) was used to continue to incubate the slices at 37°C for 10 min; then the apoptotic ICCs were observed under a fluorescence microscope (Olympus, BX53).

### 2.7 Western Blot Analysis

The total protein of colon tissues was extracted and quantified by BCA kit (Biotechnology) as described previously (Zhu et al., 2017), the protein was separated with 10% SDS-PAGE gel. After blocking the PVDF membrane with the skimmed milk powder, added the primary antibody c-kit (#3074, CST), SCF antibody (bs-0545R, BIOSS), p-AKT (#4060; CST), AKT (#9272; CST), Nrf2 (bs-1074R, BIOSS), HO-1 (bs-23397R, BIOSS), β-actin antibody (#4970, CST), GAPDH (1:5,000; Sangon Biotech, Shanghai, China) overnight at 4°C. After that, the secondary antibody was added to incubate for 1 h. Finally, all membranes were visualized by Tanon 5,200 automatic chemiluminescence imaging analysis system. Image J software (ImageJ, 1.52v) was used to analyze the gray value of the band.

### 2.8 16S rDNA Extraction and Sequencing

All DNA samples are extracted with reference to Young’s method (Yang et al., 2021). The total DNA was measured in the PCR by LC-Bio Co., Ltd (Hang Zhou, China. The primers were shown in Supplementary Table S2). All samples were sequenced on an Illumina NovaSeq platform provided by LC-Bio.

### 2.9 Statistical Processing

All data analysis and processing used GraphPad Prism 8.0.1 software and represented the mean ± SD. To compare the difference between the groups, one-way ANOVA and Student’s t-test were used. *p* < 0.05 was accepted as statistically significant.

## 3 Results

### 3.1 Pterostilbene Alleviated Loperamide-Induced Constipation Symptoms

No mouse died during the entire experiment. Since the 7th day, we found the weight in the STC group was slightly lower than normal (*p* < 0.01), but the PTE treatment group was relieved (*p* < 0.05) (Figure 1A). According to previous studies, the time of the first black stool was used to access the whole gut transit ability (Li T. et al., 2021). Compared with the CON group, the whole gut transit time was longer in the STC group (*p* < 0.05), the number of defecation particles and the water content was reduced (*p* < 0.05); In addition, the above indicators after PTE administration could be restored to the level of the CON group (*p* < 0.05) (Figures 1C–E). These results suggested that PTE had a pronounced laxative effect on LOP-induced STC mice.

### 3.2 Pterostilbene Reduced Interstitial Cells Apoptosis and Activated the Stem Cell Factor/C-Kit Pathway

**FIGURE 2:**
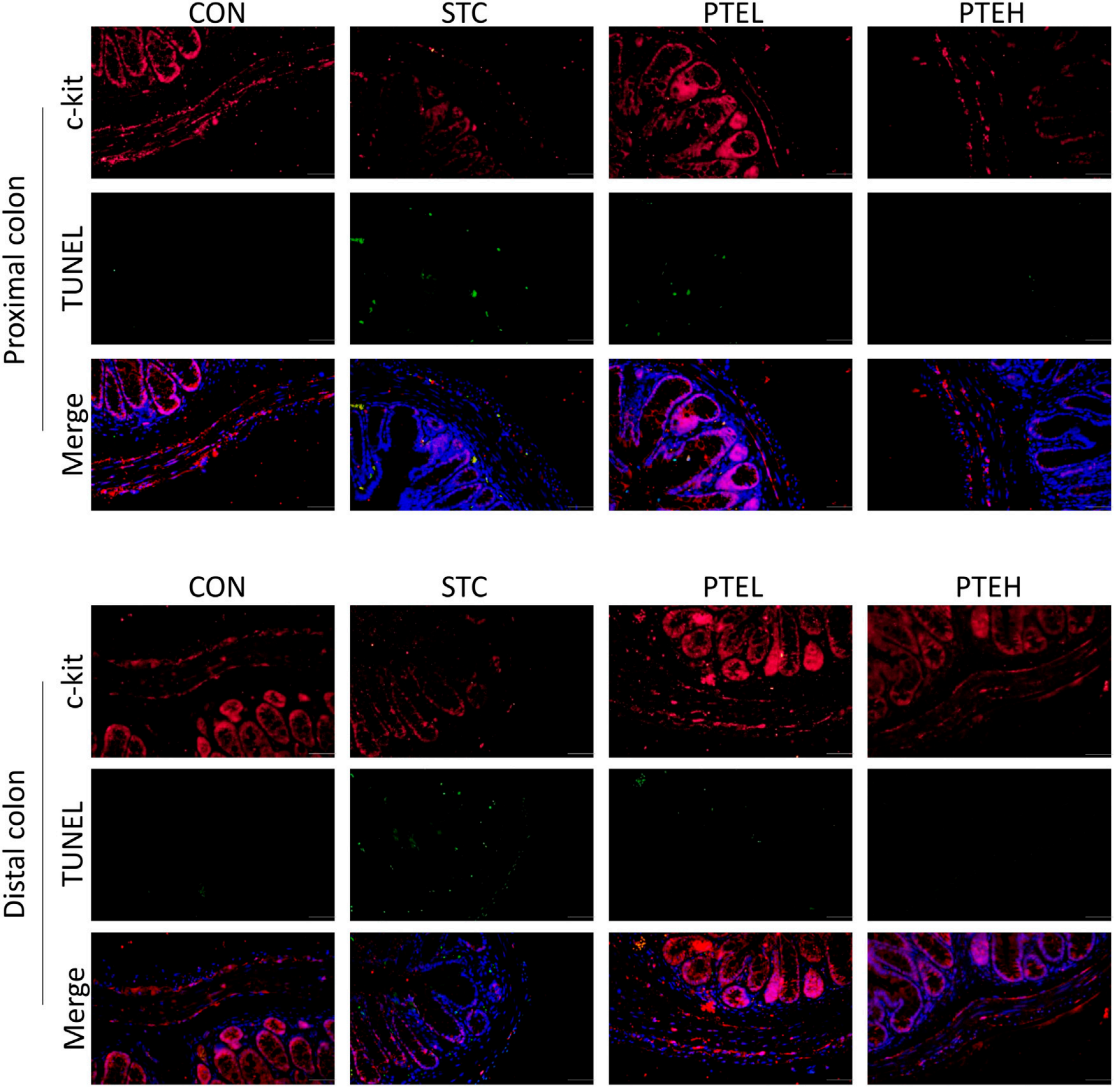
Images of cross-sections showing the alteration of the apoptosis of interstitial cells of Cajal (ICCs) in the colon. c-kit positive ICCs were stained in red and TUNEL positive ICCs were stained in green, cell nuclei were labeled with DAPI (blue). (*n* = 3 in each group). Scale bar = 100 μm.

**FIGURE 3:**
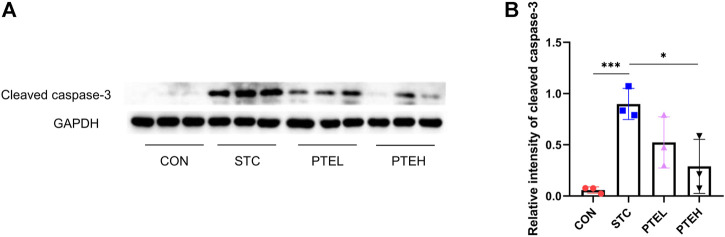
Protein expression of cleaved caspase-3 in colon tissues **(A)**. **(B)**The quantitative analysis was calculated and shown in **(B)**. *n* = 3. *p < 0.05, ***p < 0.01.

**FIGURE 4:**
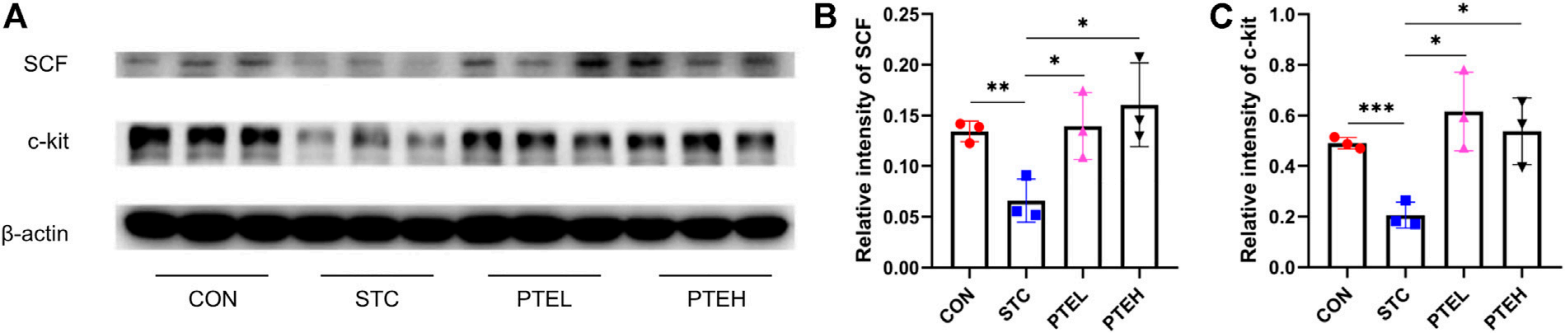
Expressions of c-kit and SCF protein in colon tissues. **(A)** The expression levels of c-kit and SCF were examined by western blot. Protein quantification of the results was shown in **(B,C)**. *n* = 3.*p < 0.05, **p < 0.01, ***p < 0.01.

TUNEL and cleaved caspase three are often used as markers of apoptosis (Wang D. et al., 2021). Therefore, TUNEL and the expressions of cleaved caspase three in each group were detected by immunofluorescence staining and western blot, respectively. There were a large number of TUNEL + cells and a small amount of c-kit + cells in both the distal and proximal colon tissues of the STC group. However, the PTE treated groups showed significantly lower numbers of TUNEL-positive cells and more c-kit + cells than the STC group (Figure 2). Furthermore, the western blot results showed that cleaved caspase-3 protein expression was significantly higher in the STC group than in the PTE treated groups. In summary, these results show that PTE reduced LOP-induced slow bowel movements by inhibiting ICCs apoptosis (Figure 3). Western blot results also showed that the expression of c-kit and SCF in the colon tissue of the STC group was significantly lower than that in the CON group (*p* < 0.01); PTE treatment could restore the expression levels of the above proteins (*p* < 0.05) (Figure 4).

### 3.3 Pterostilbene Targets the Anti-oxidative Stress Effect

**FIGURE 5:**
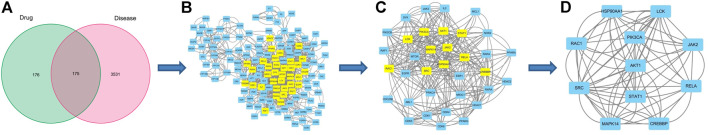
Identification of PTE-STC target genes and construction of the PPI network. **(A)** PTE target gene prediction for STC treatment. The 175 genes were considered the molecular targets of PTE against STC. **(B)** The PPI network of PTE-STC targets constructed by Cytoscape. **(C)** The PPI network of significant proteins extracted from **(B)**; **(D)** The PPI network of core targets for STC treatment obteined from **(C)**.

We found 351 potential targets for PTE and 3706 STC disease target genes (Figure 5A); As shown in Figure 5A, the 175 target genes were the intersection of STC and PTE targets. Next, we constructed a PPI network of possible target genes involved in the protective effect of PTE against STC through STRING and the network was further visualized with Cytoscape software (Figure 5B). Among these potential protein targets, we found that hub genes, such as PIK3CA, SRC, AKT1, CREBBP, RELA, HSP90AA1, RAC1, LCK, JAK2, STAT1, and MAPK14 might play a critical role in the biological activity in the PTE treatment process (Figures 5C,D).

**FIGURE 6:**
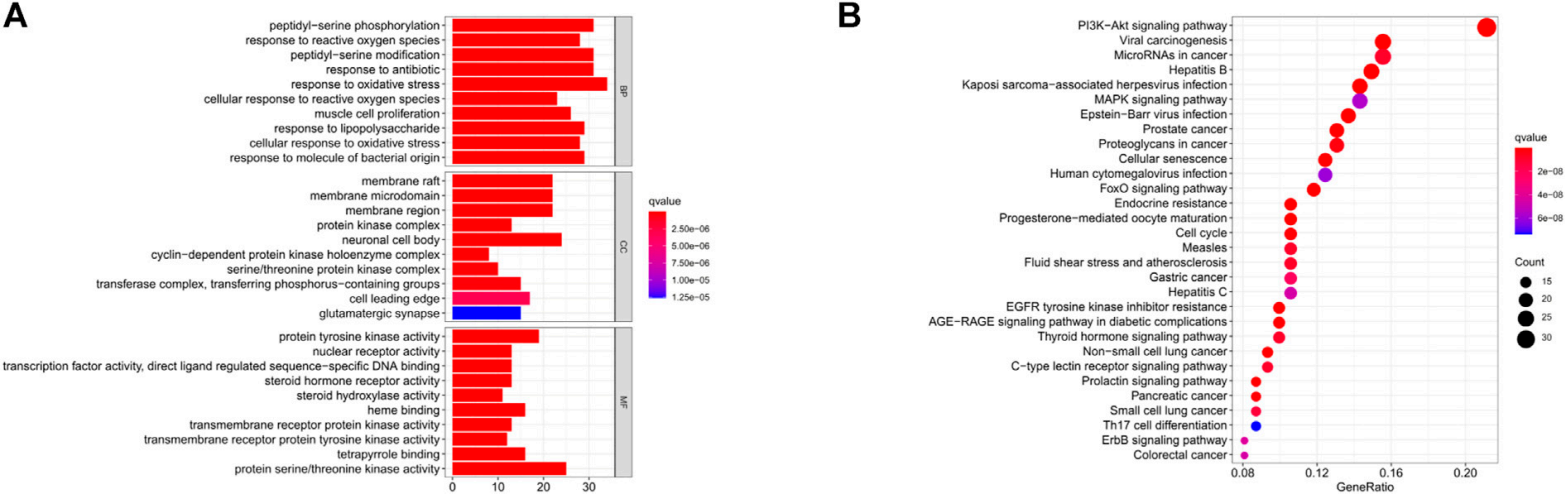
GO functional enrichment analysis **(A)** and KEGG enrichment analysis of the PTE-STC intersection target gene **(B)**.

In order to further clarify the potential mechanism of the influence of PTE on STC, a total of 175 candidate targets were analyzed by GO and KEGG pathway enrichment analysis. GO analysis indicated that PTE might improve the intestinal motility damage induced by LOP through response to reactive oxygen species and oxidative stress (Figure 6A). KEGG pathway enrichment analysis was shown in Figure 6B, which mainly involved cell proliferation-related pathways, such as PI3K/AKT signaling pathway.

The results of GO and KEGG analysis indicated that the underlying molecular mechanisms of PTE against STC were closely related to oxidative stress and PI3K/AKT signaling. Likewise, PPI analysis revealed that core target proteins of PTE were involved in the PI3K/AKT pathway, such as PIK3CA and AKT1. More and more studies have shown that PI3K/AKT exerts anti-oxidative stress effects by regulating Nrf2 (Fu et al., 2021) (Liu et al., 2022). Meanwhile, the PI3K/AKT signaling pathway also plays an important role in cell apoptosis and proliferation (Wang et al., 2018). Briefly, it was suggested that PTE might exert anti-STC effects by inhibiting oxidative stress and reducing ICCs apoptosis through the PI3K/AKT pathway. To test this hypothesis, *in vivo* experiments were performed to validate network analysis predictions.

**FIGURE 7:**
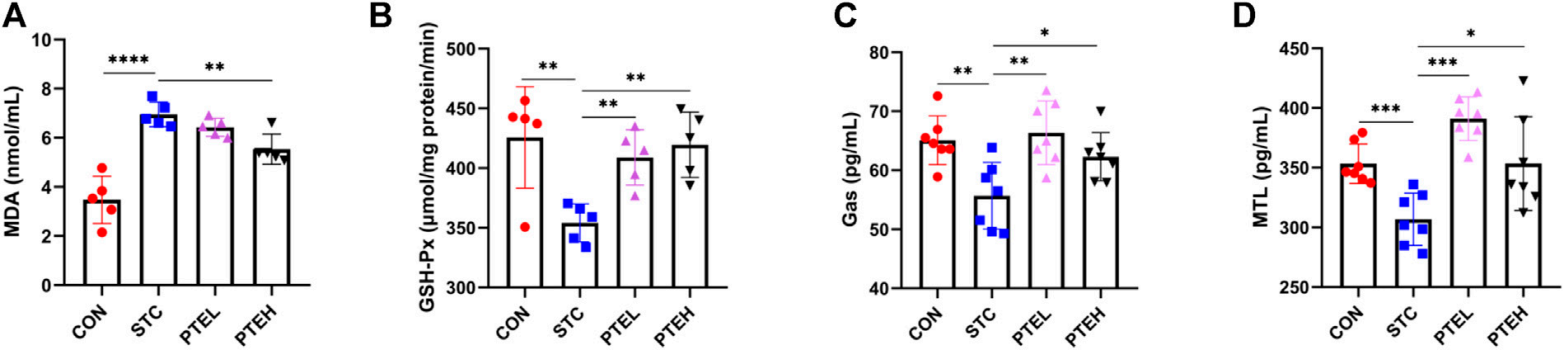
Effects of PTE on oxidative stress-related parameters and gastrointestinal hormones in serum of STC mice. **(A,B)** The malonaldehyde (MDA) level and GSH-Px were elevated in each group (*n* = 5). **(C,D)** Gas and MTL levels in serum (*n* = 7). *p < 0.05, **p < 0.01, ***p < 0.001 (Gas, Gastrin; MTL, Motilin).

Next, we measured the levels of oxidative stress marker MDA and GSH-Px, and found that LOP treatment increased oxidative product MDA in the constipated mice and depletion of antioxidant enzyme activities such as GSH-Px. To the opposite, PTE induced a significant decrease in the activity of MDA and effectively increased the activity of GSH-Px (Figures 7A,B). This indicated that LOP-induced STC mice have obvious oxidative stress, and PTE might play a laxative effect by reducing the level of oxidative stress.

### 3.4 Effects of Pterostilbene on the Expression of PI3K/AKT and Nrf2/HO-1 Pathways

**FIGURE 8:**
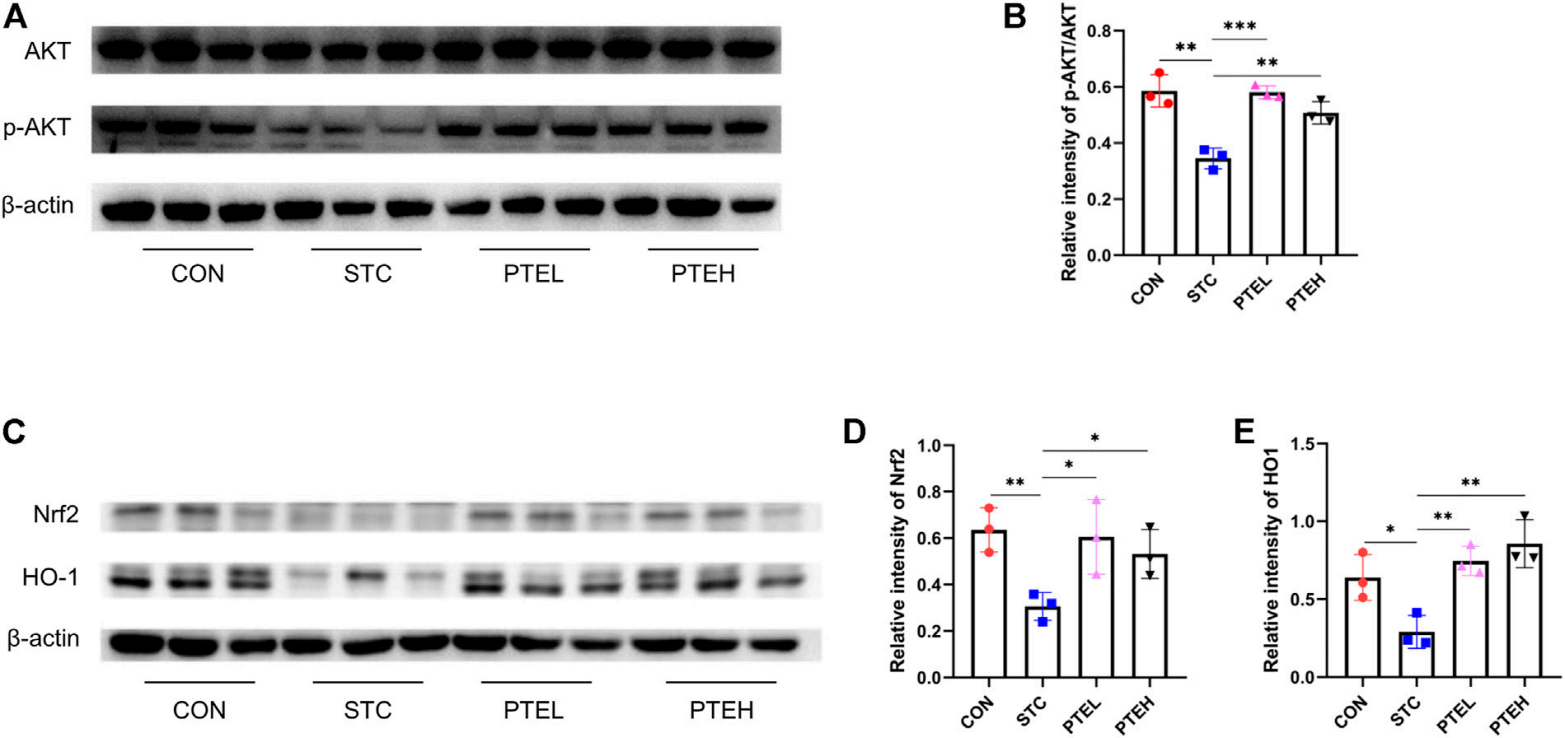
Protein expressions of Nrf2, HO-1, p-AKT, and AKT in the colon. **(A,C)** Expression levels of p-AKT, AKT, and Nrf2/HO-1 protein in the colon and the quantification analysis was calculated in **(B,D–E)**. *n* = 3. *p < 0.05, **p < 0.01, ***p < 0.001.

To further verify the downstream mechanism of PTE in STC mice, the expressions of PI3K/AKT and Nrf2/HO-1 were detected by western blot. The results showed that LOP reduced the phosphorylation of AKT and attenuated Nrf2/HO-1 expression. Compared with the STC group, PTE treatment could significantly increase p-AKT expression (Figures 8A,B). With the same trend as p-AKT, the protein expression of Nrf2/HO-1 was significantly increased in PTE treated groups. These results were consistent with predictions from network pharmacology (Figures 8C–E).

### 3.5 Pterostilbene Elevated the Levels of the Intestinal Hormone in Serum

Compared with the control group, the serum levels of motilin (MTL) and gastrin (Gas) in the STC group were significantly reduced (*p* < 0.01); However, the levels of the hormone in the PTE groups significantly increased (*p* < 0.05), indicating that PTE could alleviate constipation by regulating the level of gastrointestinal hormones (Figures 7C,D).

### 3.6 Pterostilbene Altered Diversity of Gut Microbiota in Loperamide-Induced Slow Transit Constipation Mice

**FIGURE 9:**
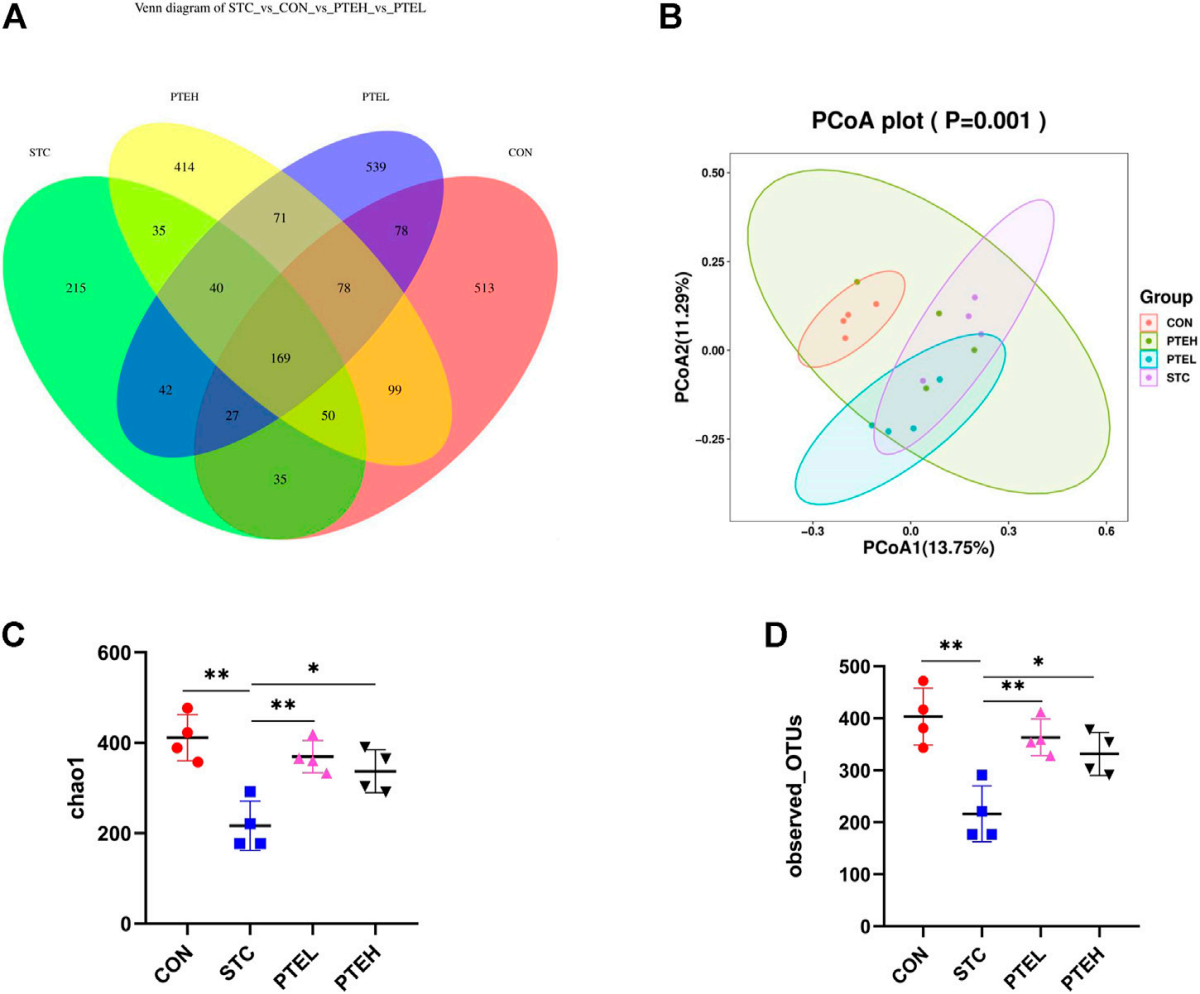
Regulation of gut microbiota structure by PTE. **(A)** The Venn diagram of different mice microflora; **(B)** The beta diversity of microbiomes in different samples, which were based on unweighted UniFrac metrics; **(C,D)** Alpha diversity indexes: **(C)** chao1 index, **(D)** Observed OTUs. (*n* = 4). *p < 0.05, **p < 0.01. (OTUs, operational taxonomic units; PCoA, principal co-ordinates analysis).

Based on the operational taxonomic units (OTUs) number, the results of the Venn diagram showed that a total of 2,405 OTUs were observed. The overlapping parts of the four groups represented the common OTUs between the groups, a total of 169 OUTs (Figure 9A).

In ecology, Alpha diversity was usually used to measure the diversity of flora within an individual (Ren et al., 2017). The fecal microbial community chao1 and observed OTUs index of mice in the STC group were lower than those in the CON group. After PTE treatment, the microbial diversity of STC mice was restored (*p* < 0.05) (Figures 9C,D). By analyzing the Beta diversity index to compare the similarity of microbial composition between groups, principal coordinate analysis (PCoA) was used. Compared with the CON group, the species composition in the STC model group was quite different, the composition of that restored in the PTE groups. The results showed that PTE could improve the diversity of intestinal microflora in mice with STC (Figure 9B).

**FIGURE 10:**
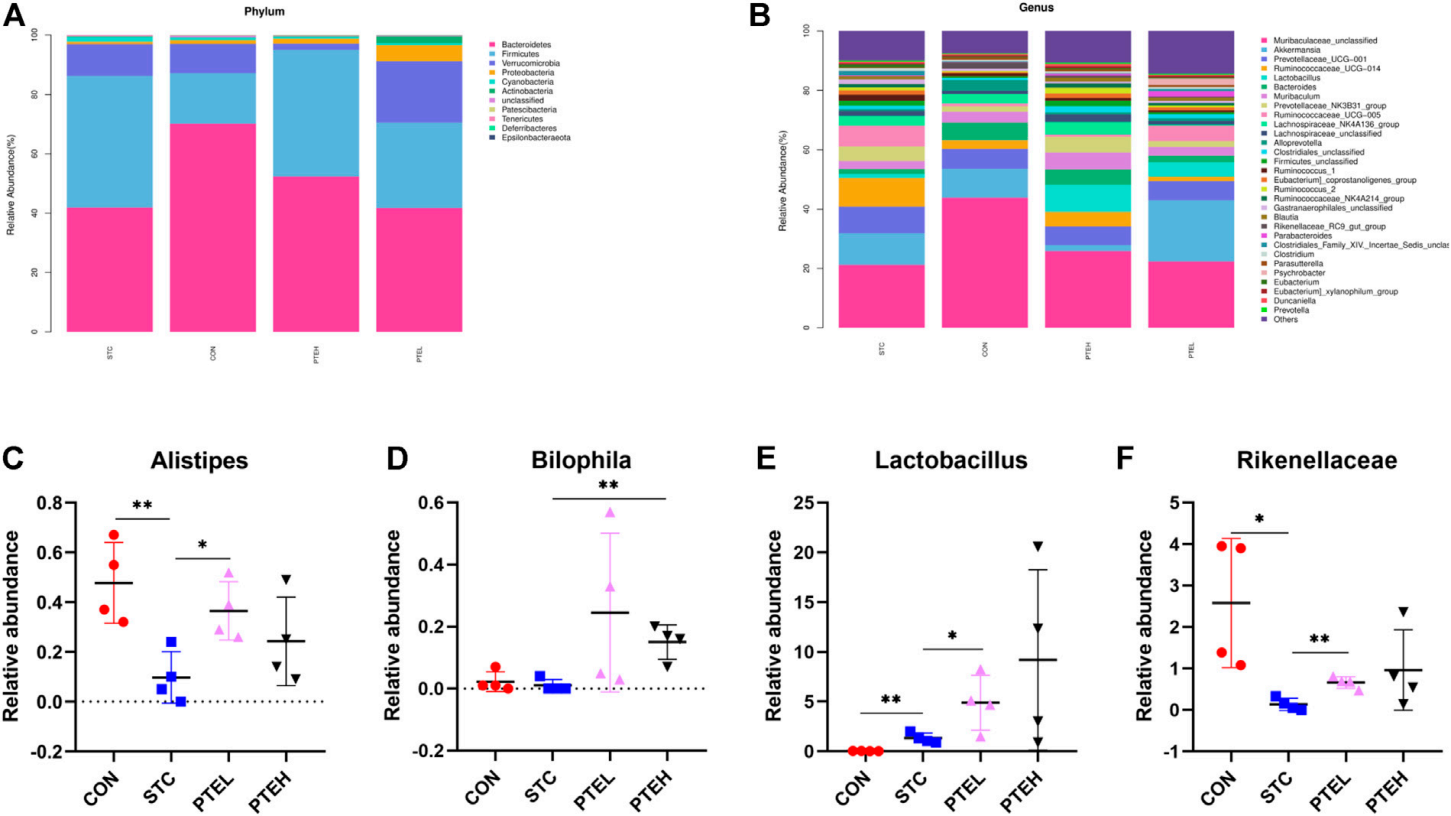
The taxonomic composition of gut microbiota in mouse feces between different groups. **(A,B)** Relative abundance of microbial communities. **(C–F)** Relative abundance of main genus in different groups: **(C)** Alipipes; **(D)** Bilophila; **(E)** Lactobacillus; **(F)** Rikenellaceae. (*n* = 4). *p < 0.05, **p < 0.01.

Figure 10A showed a columnar cluster diagram of the species composition classification of each group at the phylum level. Comparing the changes in abundance between the groups, it was found that due to the intervention of LOP, the relative abundance of Firmicutes in the STC group was higher than that in the CON group, while the relative abundance of Bacteroidetes was significantly reduced; Compared with the CON group, the relative abundance of Firmicutes in the PTE low-dose group and the PTE high-dose group increased to varying degrees, and the relative abundance of Bacteroidetes decreased, but the difference was not significant (*p* > 0.05).

At the genus level, we observed that Muribaculaceae, Akkermansia, and Prevotellaceae were the dominant bacteria (Figure 10B). Compared with the CON group, PTE intervention could increase the abundance of Lactobacillus and Bilophila (*p* < 0.05) (Figures 10D,E). Alipipes and Rikenellaceae was found higher abundance in the STC group than that in the CON group, but restored after PTE treatment (Figures 10C,F). These results indicated that the changes in the intestinal microbial structure caused by LOP in the pathogenesis of STC in mice might be reversed by PTE treatment.

## 4 Discussion

In this study, our data evaluated the laxative effect of PTE on LOP-induced constipation in mice. Next, the mechanism was predicted by network pharmacology, and PTE might play an anti-oxidative stress role through the PI3K/AKT pathway, regulate the apoptosis of ICCs, thereby improving the symptoms of constipation. *In vivo* experiments, we verified the above speculation.

Stool parameters, including stool volume, water content, and total intestinal transit time, were considered to be important factors in evaluating constipation symptoms and drug efficacy (Ren et al., 2017). We found that the stool parameters and total intestinal transit time were significantly reduced after taking LOP. Moreover, these changes could be significantly restored by PTE treatment. Our study demonstrated that PTE had a laxative effect on LOP-induced STC mice. MTL and Gas are important hormones that regulate intestinal motility (Suo et al., 2014). In this study, LOP decreased the release of MTL and Gas, but PTE treatment increased the production of hormone in a dose-dependent manner. It was suggested that PTE may alleviate the symptoms of constipation by increasing the levels of MTL and Gas.

The role of ICCs in the development of STC is increasingly appreciated, and colonic motility is significantly slowed in the absence of ICCs (Yin et al., 2018). Apoptosis is a common method of cell reduction. There is evidence that ICCs apoptosis is observed in the colon of a majority of STC patients (Zheng et al., 2021), and apoptotic ICCs can be identified by TUNEL immunostaining. In this experiment, we used c-kit immunolabeled colon sections and TUNEL staining to detect apoptotic ICCs, and found that apoptotic ICCs increased in STC group, while the number of apoptotic ICCs decreased in PTE groups. The SCF/c-kit pathway is the basic guarantee to maintain the number of ICCs (Wang et al., 2021b). PTE administration could enhance the expression of SCF/c-kit pathway and reduce the apoptosis of ICCs. It was suggested that PTE had an anti-apoptotic effect on ICCs in mouse colon and further promoted intestinal transit.

So what is the mechanism that causes PTE to reduce ICCs apoptosis? We further found that PI3K/AKT signaling might reduce ICCs apoptosis by mediating downstream Nrf2/HO-1 signaling to play an anti-oxidative stress role through network pharmacology analysis.

The breakdown of the oxidative-antioxidant balance will lead to mitochondrial dysfunction, lipid peroxidation, or apoptosis. Previous studies have shown that oxidative stress has an important role in ICCs apoptosis (Wang et al., 2021b). Meanwhile, elevated levels of oxidative stress and significant ICCs loss were observed in both STC patients and LOP-induced constipation animal models (Gibbons et al., 2009) (Lee et al., 2020). Therefore, reducing oxidative stress-induced ICCs apoptosis may be a therapeutic target for STC. MDA is a biomarker of oxidative stress, and GSH-Px is an important reactive oxygen species scavenging enzyme that maintains redox balance in the body (Hajji et al., 2020). According to our results, in the STC group, the MDA content increased, while the GSH-Px activity decreased. In contrast, treatment with PTE reversed the above changes. In addition, LOP-induced apoptosis of ICCs in colon was significantly alleviated after PTE intervention treatment. These results suggested that PTE may protect ICCs from oxidative stress-induced apoptosis.

More and more studies have found that the PI3K/AKT pathway regulates cellular oxidative stress by enhancing the expression of Nrf2/HO-1 and exerts cytoprotective effects (Koundouros and Poulogiannis, 2018) (Gao et al., 2020). Nrf2 is a transcription factor that regulates the expression of oxidative stress-related proteins and enzymes involved in metabolism and detoxification, such as HO-1 (Loboda et al., 2016). Under conditions of oxidative stress, Nrf2 activates transcription to generate various antioxidant enzymes in the cytoplasm, including HO-1, CAT, and GSH-Px (Zhuang et al., 2019) (Wen et al., 2018). Previous studies have elucidated that Nrf2 plays a key role in protecting ICCs from oxidative stress (Wang et al., 2021c). Activation of Nrf2 can reduce oxidative stress and reduce apoptosis in ICCs. In our study, apoptosis of ICCs in the colon of STC mice and reduction of Nrf2/HO-1 pathway were observed, resulting in slower colonic transit in mice. Consistent with our data, PI3K/AKT signaling was significantly reduced in STC mice with a large number of apoptotic ICCs, whereas PTE treatment upregulated AKT phosphorylation levels, promoted Nrf2/HO-1 expression, and reduced the number of apoptotic ICCs. These results suggested that PTE could enhance the Nrf2/HO-1 pathway through the PI3K/AKT pathway to protect ICCs from oxidative stress injury and reduce apoptosis, thereby alleviating intestinal motility disorders.

In recent years, many studies pointed out that intestinal flora was closely related to the occurrence and development of STC (De Vadder et al., 2018) (Müller et al., 2020). In our study, the diversity of microbiota in constipated mice was reduced, and PTE had a greater impact on the richness and diversity of the intestinal microbial community in STC mice, especially in the PTEH group. PTE intervention could increase the abundance of Lactobacillus and Bilophila. Lactobacillus is a well-known beneficial intestinal bacteria and Bilophila is essential for the production of hydrogen sulfide in the intestine (Hanson et al., 2021). The gaseous transmitter hydrogen sulfide promoted the proliferation of ICCs through phosphorylation of AKT protein kinase (Huang et al., 2010). The abundance of Alipipes and Rikenellaceae in the STC group was much lower, but recovered after PTE treatment. According to reports, Alisipes and Rikenellaceae are all butyric acid-producing bacteria, butyric acid and other SCFAs have obvious laxative activity and stimulate ICCs proliferation (Wu et al., 2020) (Xu et al., 2020). Thus, the laxative effect of PTE may be co-regulated by all these mechanisms. On the other hand, this study also has some limitations. We did not further explore the relationship between ICCs and colonic contractile activity, and will be the focus of future research.

## 5 Conclusion

In this study, a combination of network pharmacology predictive analysis and experimental verification was used to explore the laxative effect of PTE on LOP-induced STC and its possible mechanism. PTE might play a laxative effect by inhibiting oxidative stress-induced apoptosis of ICCs through the PI3K/AKT/Nrf2 signaling pathway. The results of animal experiments also verified the results predicted by network analysis. In addition, PTE treatment restored the disordered gut microbes in STC mice. Our results provided experimental and theoretical evidence and suggested PTE as a promising drug for STC therapy.

## Data Availability

The datasets presented in this study can be found in online repositories. The names of the repository/repositories and accession number(s) can be found below: https://www.ncbi.nlm.nih.gov/sra/PRJNA820465.
