# Supplementary material for: Based on Network Pharmacology and Gut Microbiota Analysis to Investigate the Mechanism of the Laxative Effect of Pterostilbene on Loperamide-Induced Slow Transit Constipation in Mice
Source: Front Pharmacol. 2022 May 16;13:913420. doi: 10.3389/fphar.2022.913420 (PMC9148975; doi:10.3389/fphar.2022.913420)
Supplement: Supplementary file 1 [file Table1.DOCX]

Table S1: Databases and platforms used for the network pharmacology analysis

| Database | Sources |
| --- | --- |
| PharmMapper | https://[www.lilab-ecust.cn/pharmmapper/](http://www.lilab-ecust.cn/pharmmapper/) |
| STITCH | https://stitch.embl.de/ |
| SwissTargetPrediction | https://www.swisstargetprediction.ch/ |
| Chembl | https://www.library.ucsb.edu/node/6611 |
| UniPort | https://www.uniprot.org |
| PharmGkb | https://www.pharmgkb.org/ |
| OMIM | https://www.omim.org |
| GeneCards | https://www.genecards.org |
| Drugbank | http://www.drugbank.ca/ |
| TTD | http://bidd.nus.edu.sg/group/cjttd/ |
| STRING | http://stringdb.org |

Table S2: The primer sequences of 16s rDNA

| Forward 5’-3’ | Reverse 5’-3’ |
| --- | --- |
| CCTACGGGNGGCWGCAG | GACTACHVGGGTATCTAATCC |
