# Supplementary material for: Based on Network Pharmacology and Gut Microbiota Analysis to Investigate the Mechanism of the Laxative Effect of Pterostilbene on Loperamide-Induced Slow Transit Constipation in Mice
Source: Front Pharmacol. 2022 May 16;13:913420. doi: 10.3389/fphar.2022.913420 (PMC9148975; doi:10.3389/fphar.2022.913420)
Supplement: Supplementary file 2 [file DataSheet1.PDF]

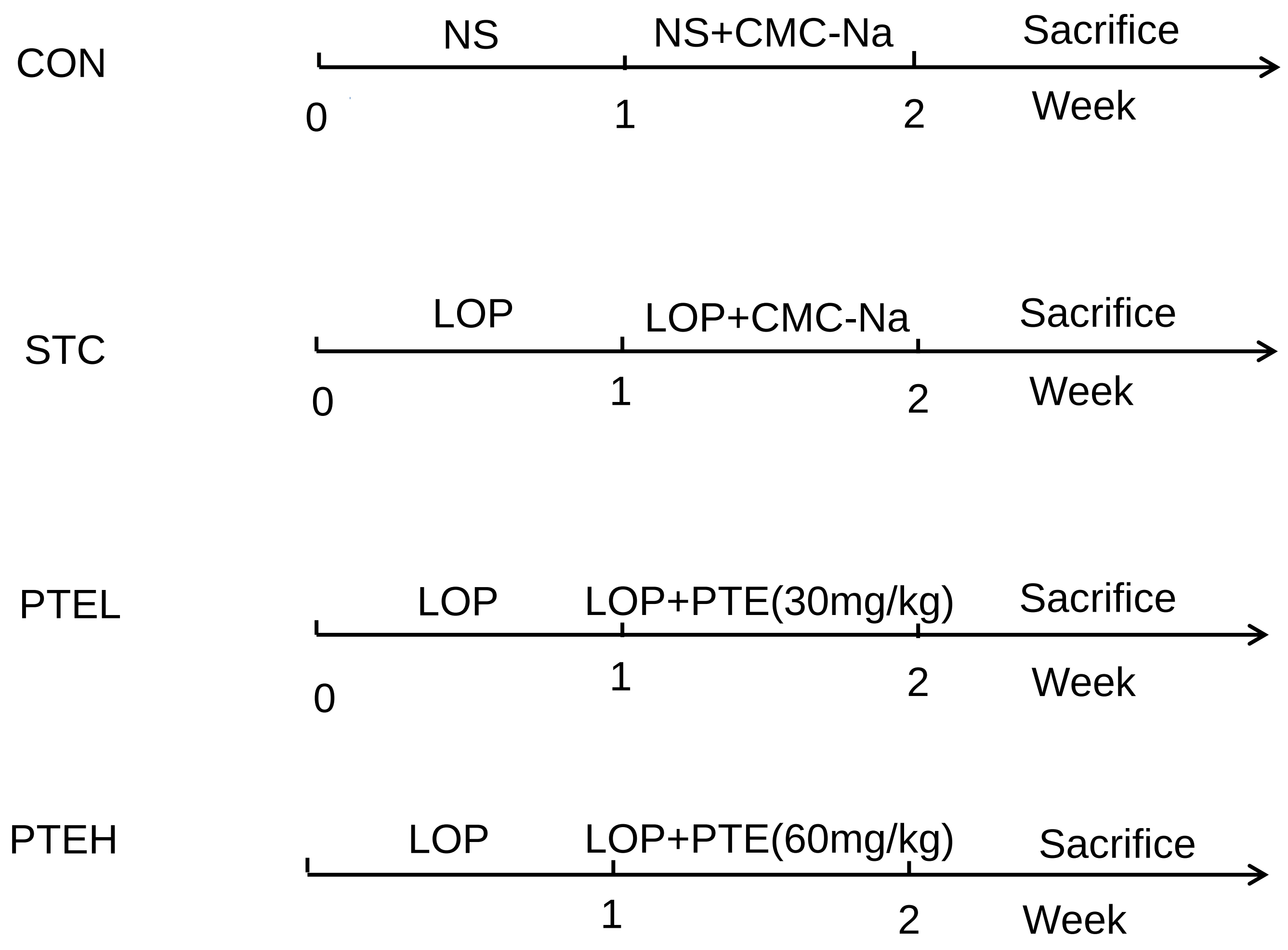

Figure S1 Establishment of STC mouse model and PTE administration
(LOP: loperamide; STC: slow transit constipation; PTE: pterostilbene; NS: normal saline; CMC-Na: carboxymethylcellulose sodium)

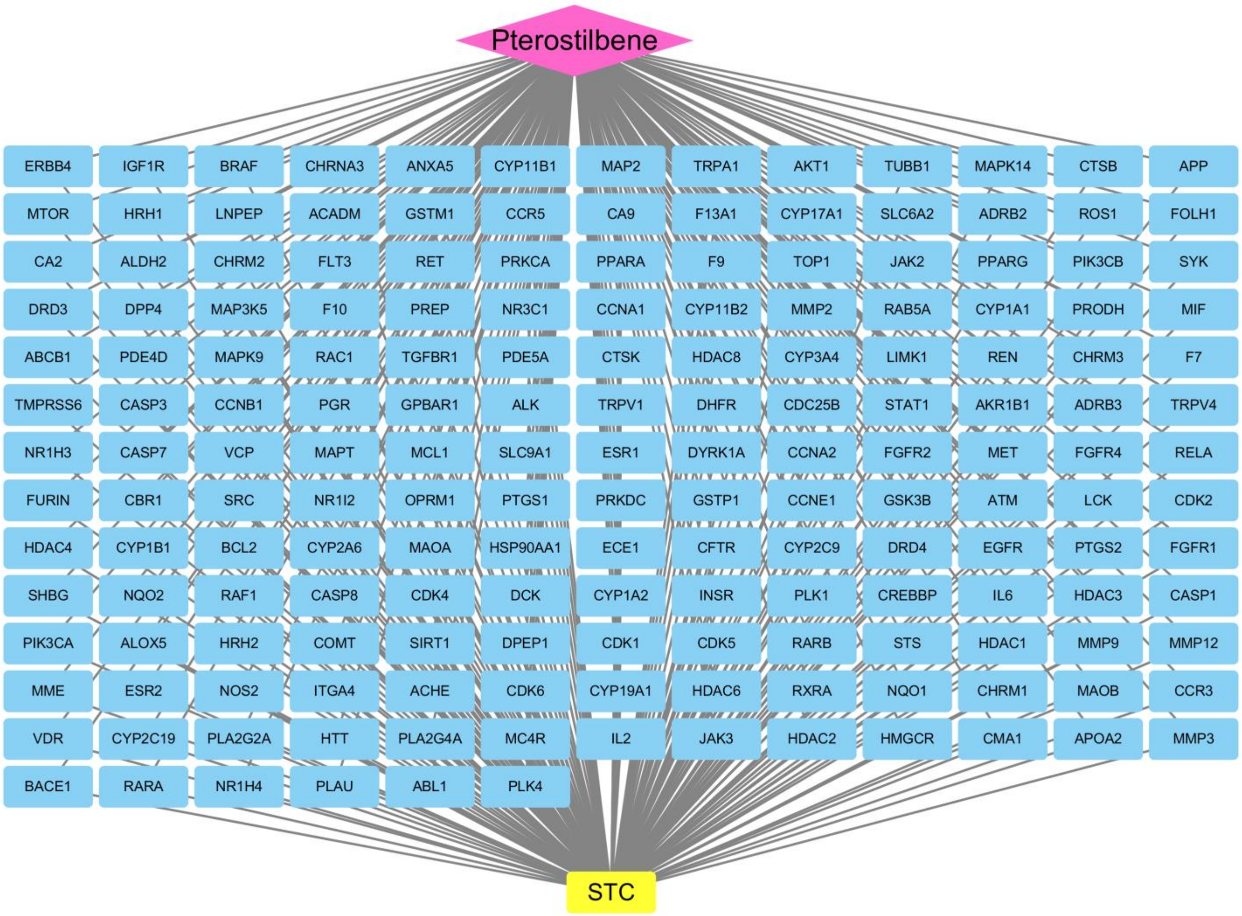

Figure S2 Target network of "PTE-STC-targets" was constructed using Cytoscape software

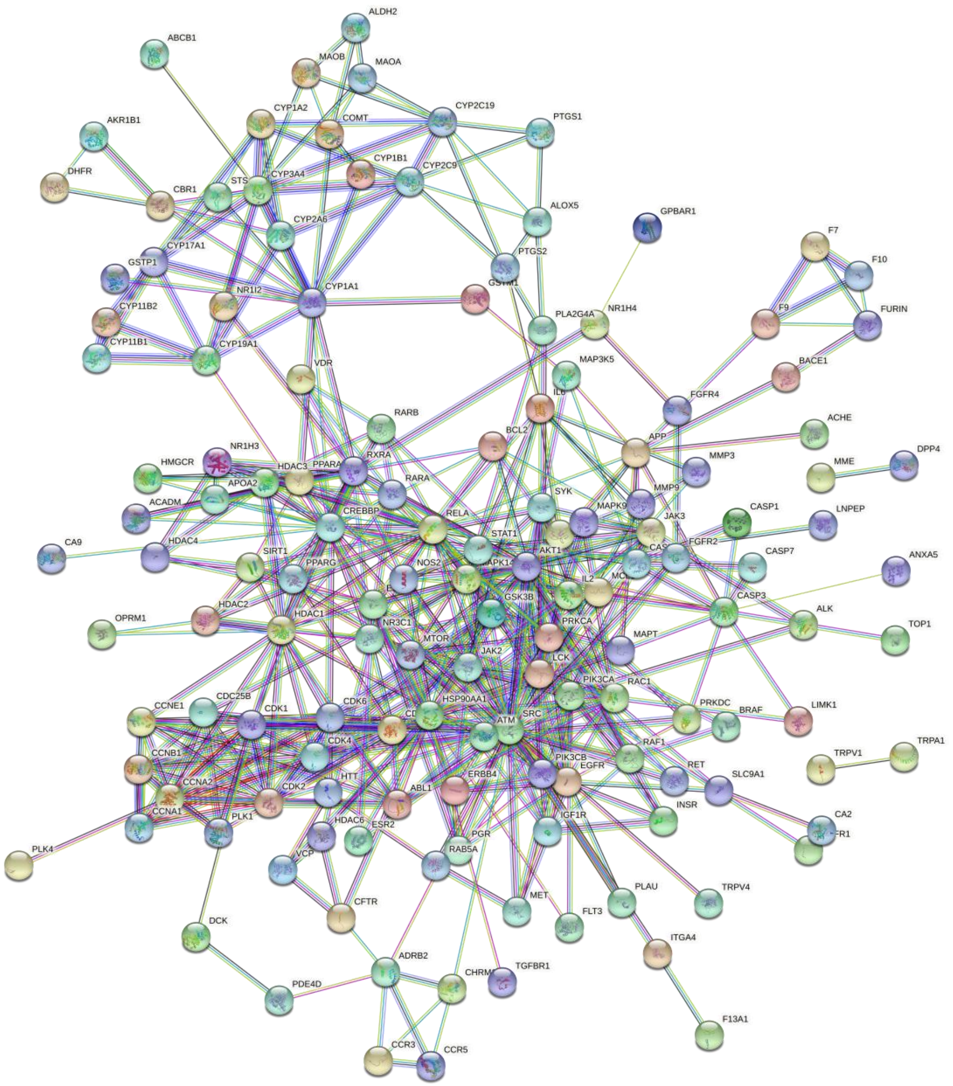

Figure S3 Protein–protein interaction (PPI) networks constructed in STRING
